# Electrical and Optical Properties Depending on the Substitution Position of a Novel Indolocarbazole Dimer

**DOI:** 10.3390/ma18092058

**Published:** 2025-04-30

**Authors:** Jiyun Kim, Suhyeon Jeong, Sangwook Park, Saeyoung Oh, Kiho Lee, Soonhang Lee, Jihoon Lee, Hayoon Lee, Jongwook Park

**Affiliations:** 1 Integrated Engineering, Department of Chemical Engineering, Kyung Hee University, Yongin 17104, Republic of Korea; jiyunkimmm@khu.ac.kr (J.K.); jsh5142@khu.ac.kr (S.J.); pswook@khu.ac.kr (S.P.); tpdud5821@khu.ac.kr (S.O.); kiholee@khu.ac.kr (K.L.); kssarang1@khu.ac.kr (H.L.); 2 Department of Polymer Science and Engineering and Department of IT∙Energy Convergence (BK21 FOUR), Korea National University of Transportation, Chungju 27469, Republic of Korea; gumneuk@naver.com (S.L.); jihoonli@ut.ac.kr (J.L.)

**Keywords:** OLED, phosphorescence, host, indolocarbazole, dimer

## Abstract

Two innovative dimeric derivatives of indolo[3,2,1-jk]carbazole (ICz), named 7,7′-biindolo[3,2,1-jk]carbazole (ICzDO) and 4,4′-biindolo[3,2,1-jk]carbazole (ICzDM), have been developed. Both dimers consist of two ICz units coupled through distinct ortho and meta positions. In the solution state, ICzDO and ICzDM exhibited photoluminescence (PL) maxima at 379 nm and 391 nm, demonstrating emission in the deep-blue region. These compounds show exceptionally narrow emission spectra, characterized by full width at half maximum (FWHM) of 28 nm for ICzDO and 26 nm for ICzDM. In the film state, ICzDM exhibited a photoluminescence (PL) maximum at 428 nm, whereas ICzDO showed a red-shifted emission at 507 nm with a broad full width at half maximum (FWHM) of 87 nm, indicating significant red-shifted excimer emission characteristics. This is attributed to its aggregation-enhanced excimer emission (AEEE) characteristics. When used as host materials for red phosphorescent OLEDs, both compounds enabled efficient energy transfer. Devices using ICzDM as the host attained highly efficient external quantum efficiency (EQE) values of 13.5%, coupled with remarkable color purity represented by Commission Internationale de l’Éclairage (CIE) coordinates of (0.685, 0.314). These findings emphasize how strategic variations in linking positions of identical chromophores can markedly enhance OLED device performance, paving the way for innovative material designs in next-generation organic semiconductor technologies.

## 1. Introduction

Phosphorescent organic light-emitting diodes (PhOLEDs) are capable of utilizing both singlet (25%) and triplet (75%) excitons, thereby achieving a theoretical internal quantum efficiency of up to 100%. Significant research efforts have been dedicated to the development of various host materials aimed at enhancing the efficiency of PhOLEDs. An ideal host material should exhibit excellent charge-transporting properties and possess a higher triplet energy level than the dopant to facilitate effective exciton confinement. Additionally, high thermal and chemical stability are essential to ensure reliable device operation. Molecular frameworks such as carbazole, dibenzofuran, and triazine are commonly selected for host material design due to their advantageous electronic structures and intrinsic durability [1,2,3]. Among various host-design moieties, carbazole has been extensively utilized not only in OLEDs but also in other optoelectronic applications such as organic thin-film transistors (OTFTs) and organic photovoltaics (OPVs), due to its robust electron-donating character and outstanding resistance to thermal and chemical degradation [4,5,6]. Another promising structural variant, indolocarbazole, emerges from the fusion of two carbazole units at different positions, creating opportunities for targeted molecular design. A particularly intriguing derivative, ICz, is characterized by its distinct nitrogen-centered tripod arrangement [7,8]. This configuration, wherein three benzene units are electronically conjugated via the lone pair electrons of a central nitrogen atom, significantly enhances the charge-carrier transport properties relative to the carbazole parent structure.

In recent research, Kim et al. introduced phenylcarbazole and dibenzothiophene groups into an ICz backbone, synthesizing two novel host compounds, ICz-PCz and ICz-DBT. These derivatives exhibited high triplet energy levels exceeding 2.86 eV, positioning them as promising host candidates for green phosphorescent emitters. OLEDs fabricated with these materials as hosts delivered impressive device performances, achieving external quantum efficiencies (EQEs) as high as 23%, underscoring their applicability in advanced optoelectronic devices [9]. In 2023, the ICz core was functionalized with a diphenylsilane moiety, resulting in the novel host compound Si-IDCz, exhibiting a high triplet energy of 2.88 eV. OLED devices using Si-IDCz demonstrated blue phosphorescence with an EQE of 16.9% [10]. In a related study, T. Hatta et al. enhanced bipolar charge transport characteristics by incorporating a pyrimidine unit into the ICz structure. This modification provided balanced hole and electron transport, enabling its successful application as a host in thermally activated delayed fluorescence (TADF) OLEDs, achieving an EQE of 13.9% [11]. These findings reveal the versatility of ICz-based materials, which combine excellent thermal stability and charge transport properties, making them suitable for both phosphorescent and TADF host applications. Despite its advantageous electronic properties, ICz exhibits a higher degree of molecular planarity compared to carbazole, which can lead to undesirable aggregation-caused quenching (ACQ) in the solid state [12]. This phenomenon may result in reduced device efficiency, particularly in emissive layers. To address this issue, strategies such as incorporating aggregation-induced emission (AIE) or aggregation-enhanced excimer emission (AEEE) characteristics have been explored to suppress ACQ and improve overall device performance. AIE has proven to be an effective strategy for overcoming the common issue of ACQ, which typically arises from strong π–π stacking interactions. This mechanism, primarily attributed to the restriction of intramolecular rotations (RIR), allows for efficient luminescence in the solid state [13]. In 2011, the Tang group reported the first observation of AEEE in a pyrene derivative functionalized with a tetraphenylethene (TPE) core, a typical AIE-active structure [14]. AEEE-based materials play a key role in maintaining high emission efficiency in thin films by optimizing intermolecular distances and facilitating the formation of stable excimer states.

This work presents the synthesis and characterization of two dimer-type molecules constructed from ICz, a core known for its enhanced charge-transporting capabilities relative to carbazole. The dimers, ICzDO and ICzDM, feature linkage at different positions—ortho, which facilitates π-conjugation, and meta, which suppresses it—allowing for a comparative analysis of structure-property relationships. By introducing a moderately twisted geometry between the units, we investigated how the linking position influences photophysical behavior and device performance. Both compounds were applied as host materials in red phosphorescent OLEDs, and their optical and electroluminescent properties were systematically investigated.

## 2. Materials and Methods

### 2.1. Materials and Instrumentation

All reagents and solvents were of reagent grade and used as received with no further purification steps. Analytical thin-layer chromatography (TLC) was performed on Merck 60 F254 silica gel plates. For column chromatography, Merck 60 silica gel (230–400 mesh, Burlington, MA, USA) was used as the stationary phase. ^1^H NMR spectra were obtained in dimethyl sulfoxide-d6 (DMSO-d6) on a JNM-ECZ400S/L1 spectrometer (JEOL, Tokyo, Japan) at room temperature. High-resolution mass spectrometry (HRMS) was conducted via electron ionization (EI) on a JMS-700, 6890 Series mass spectrometer (JEOL, Tokyo, Japan). Ultraviolet–visible (UV–vis) absorption spectra measured with a UV-1900i UV/Vis/NIR spectrophotometer (Shimadzu, Kyoto, Japan). Photoluminescence (PL) spectra were measured using a PerkinElmer LS55 spectrofluorometer equipped with a xenon flash lamp (PerkinElmer, Inc., Waltham, MA, USA). Absolute photoluminescence quantum yields (PLQYs) were determined using a Hamamatsu Quantaurus-QY C11347 system (Hamamatsu Photonics, Shizuoka-ken, Japan). Transient photoluminescence lifetimes were measured using a Quantaurus-Tau fluorescence lifetime spectrometer (Hamamatsu Photonics, Shizuoka-ken, Japan). Density functional theory (DFT) calculations for 2TRZ-P-ICz and 2TRZ-TP-ICz were performed using the B3LYP-D3 functional and def2-TZVPP basis set as implemented in the ORCA software suite (v5.0.4) [15]. Thermal properties including glass transition temperature (T_g_), melting temperature (T_m_), and crystallization temperature (T_c_) were investigated by differential scanning calorimetry (DSC) under a nitrogen atmosphere using a DSC 26 instrument (TA Instruments, New Castle, DE, USA). Decomposition temperatures (T_d_) were determined by thermogravimetric analysis (TGA) on an SDT Q600 system (TA Instruments, New Castle, DE, USA), with samples heated to 700 °C at a rate of 10 °C/min. Ultraviolet photoelectron spectroscopy (Riken Keiki AC-2, Nara, Japan) was employed to determine the highest occupied molecular orbital (HOMO) levels, while the lowest unoccupied molecular orbital (LUMO) levels were estimated from the HOMO values and optical band gaps. Thin films of the synthesized compounds were deposited under vacuum to a thickness of 50 nm at a rate of 1 Å/s and a base pressure of 10^−6^ Torr. For electroluminescent (EL) device fabrication, all organic layers were deposited under identical vacuum conditions and rates, covering an area of 4 mm^2^. LiF and Al electrodes were sequentially deposited under identical vacuum parameters. The current density–voltage–luminance (J–V–L) characteristics of the fabricated EL devices were measured using a Keithley 2400 source meter (Tektronix, Cleveland, OH, USA), and luminance intensities were recorded using a Minolta CS-1000A spectroradiometer (Konica Minolta, Tokyo, Japan). All devices were stored in a glovebox to minimize exposure to ambient moisture and oxygen.

### 2.2. Synthesis and Characterization

#### 2.2.1. Synthesis of 9-(2,6-Dibromophenyl)-9H-carbazole, (**1**)

In a 100 mL round-bottom flask, carbazole (1.00 g, 5.98 mmol) and 1,3-dibromo-2-fluorobenzene (1.52 g, 5.98 mmol) were combined with cesium carbonate (Cs_2_CO_3_) (5.85 g, 5.90 mmol) containing 30 mL of anhydrous N,N-dimethylformamide (DMF). The suspension was heated at 160 °C under reflux with vigorous stirring for 3 h. After cooling, the reaction mixture was partitioned between dichloromethane and deionized water. The organic layer was isolated, dried over anhydrous magnesium sulfate (MgSO_4_), filtered, and the solvent was evaporated under vacuum. The crude material was subjected to chromatographic purification over silica gel using a dichloromethane/hexane (2:8, *v*/*v*) eluent system. The purified product was obtained as a white solid in a yield of 61.0% (1.46 g). ^1^H NMR (400 MHz, DMSO-d6) δ 8.21 (d, J = 8.0 Hz, 2H), 7.97 (d, J = 8.2 Hz, 2H), 7.55–7.45 (t, J = 8 Hz, 1H), 7.38 (t, J = 8.0 Hz, 2H), 7.26 (t, J = 8.0, 7.2, 1.0 Hz, 2H), 6.91 (d, J = 8.1 Hz, 2H).

#### 2.2.2. Synthesis of 7,7′-Biindolo[3,2,1-jk]carbazole (ICzDO)

In a 100 mL round-bottom flask, a mixture of 9-(2,6-dibromophenyl)-9H-carbazole (1.00 g, 2.49 mmol), palladium(II) acetate (Pd(OAc)_2_) (0.08 g, 0.37 mmol), triphenylphosphine (PPh_3_) (0.23 g, 0.87 mmol), potassium carbonate (K_2_CO_3_) (1.72 g, 12.4 mmol), and ammonium bromide (NH_4_Br) (0.24 g, 2.49 mmol) was dissolved in 20 mL of dimethylacetamide (DMA). The reaction mixture was stirred and heated at 160 °C under reflux for 2 h. After completion, the mixture was extracted with dichloromethane and deionized water. The organic phase was dried over anhydrous MgSO_4_, filtered, and concentrated under reduced pressure. The resulting crude product was purified by silica gel column chromatography using a dichloromethane/hexane mixture (2:8, *v*/*v*) as the eluent, affording the desired compound in a yield of 13.4% (0.16 g). ^1^H NMR (400 MHz, DMSO-d6) δ 8.63 (d, J = 8.0 Hz, 2H), 8.32 (d, J = 7.4 Hz, 2H), 8.10 (d, J = 7.3 Hz, 2H), 8.03–7.99 (d, J = 4.0 Hz, 2H), 7.79 (d, J = 8.0 Hz, 2H), 7.65 (m, 6H), 7.00 (t, J = 8.0 Hz, 2H), 6.73 (t, J = 8.0 Hz, 2H). HRMS (EI-MS, *m*/*z*): [M+] calc’d for C36H20N2, 480.1626; found, 480.1630. Elemental analysis calculated (%) for C_36_H_20_N_2_: C, 89.98; H, 4.19; N, 5.83; found: C 88.53, H 4.20, N 5.70.

#### 2.2.3. Synthesis of 9-(2,3-Dibromophenyl)-9H-carbazole, (**2**)

In a 100 mL round-bottom flask, carbazole (0.33 g, 1.97 mmol) and 1,2-dibromo-3-fluorobenzene (0.50 g, 1.96 mmol) were combined with Cs_2_CO_3_ (1.92 g, 5.90 mmol) containing 30 mL of anhydrous N,N-dimethylformamide (DMF). The suspension was heated at 160 °C under reflux with vigorous stirring for 3 h. After completion, the reaction mixture was partitioned between dichloromethane and deionized water. The organic layer was isolated, dried over anhydrous MgSO_4_, filtered, and the solvent was evaporated under vacuum. The crude material was subjected to chromatographic purification over silica gel using a dichloromethane/hexane (2:8, *v*/*v*) eluent system. The purified product was obtained as a white solid in a 72.2% yield (0.57 g). ^1^H NMR (400 MHz, DMSO-d6) δ 8.21 (d, J = 8.0 Hz, 2H), 8.00 (d, J = 4.0 Hz, 1H), 7.63 (m, 2H), 7.38 (t, J = 8.0 Hz, 2H), 7.26 (t, J = 8.0Hz, 2H), 6.99 (d, J = 8.0 Hz, 2H).

#### 2.2.4. Synthesis of 4,4′-Biindolo[3,2,1-jk]carbazole (ICzDM)

In a 100 mL round-bottom flask, a mixture of 9-(2,3-dibromophenyl)-9H-carbazole (1.00 g, 2.49 mmol), Pd(OAc)_2_ (0.08 g, 0.37 mmol), PPh_3_ (0.23 g, 0.87 mmol), K_2_CO_3_ (1.72 g, 12.4 mmol), and NH_4_Br (0.24 g, 2.49 mmol) was dissolved in 20 mL of DMA. The reaction mixture was stirred and heated at 160 °C under reflux for 2 h. After the reaction had finished, the mixture was cooled and extracted with dichloromethane and deionized water. The organic phase was separated, dried over anhydrous MgSO_4_, filtered, and concentrated under reduced pressure. The crude product was purified by column chromatography on silica gel using a 2:8 (*v*/*v*) mixture of dichloromethane and hexane, affording the target compound in 24% yield (0.19 g). ^1^H NMR (400 MHz, DMSO-d6) δ 8.49 (d, J = 8.0 Hz, 2H), 8.40 (d, J = 8.1, 0.9 Hz, 2H), 8.24 (d, J = 8.0 Hz, 2H), 8.01 (d, J = 4.0 Hz, 2H), 7.85 (t, J = 8.0 Hz, 2H), 7.68–7.62 (m, 4H), 7.43–7.40 (t, J = 8.0 2H), 7.17 (t, J = 8.0 Hz, 2H), 6.89 (d, J = 7.5 Hz, 2H). HRMS (EI-MS, *m*/*z*): [M+] calc’d for C36H20N2, 480.1626; found, 480.1624 Elemental analysis calculated (%) for C_36_H_20_N_2_: C, 89.98; H, 4.2; N, 5.83; found: C 89.997, H 5.478, N 5.519.

## 3. Results and Discussion

### 3.1. Molecular Design, Synthesis, and Characterization

**Scheme 1:**
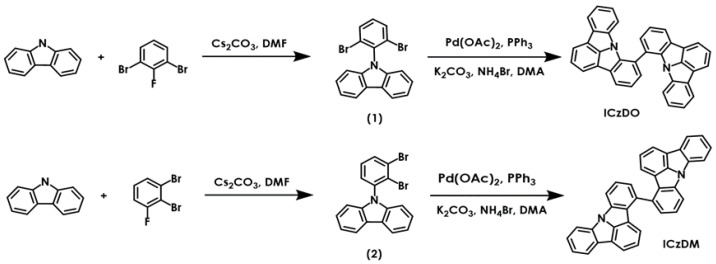
Synthetic routes of the target compounds.

**Figure 1:**
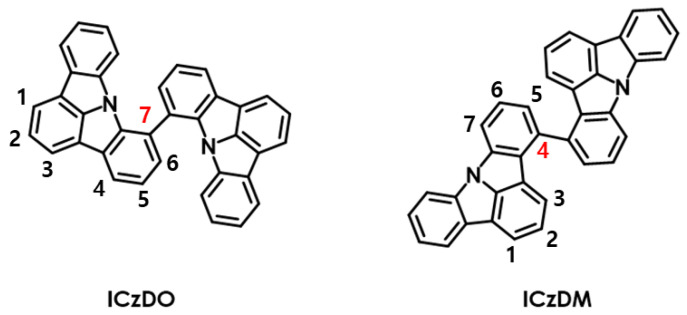
Chemical structures of the synthesized compounds ICzDO and ICzDM.

In this study, two novel dimer-type OLED materials, ICzDO and ICzDM, were synthesized by linking ICz units through ortho and meta positions, respectively, to investigate the influence of linkage geometry on their properties (Figure 1). The ICz core, consisting of two rigidly fused carbazole units, effectively suppresses molecular vibrations, which contributes to its narrow emission bandwidth. This structural rigidity enables a small Stokes shift, facilitating the design of chromophores with high color purity and minimal energy loss during emission. However, due to the high planarity of the ICz core, para-substitution leads to extended intramolecular conjugation, resulting in emission at significantly longer wavelengths. Such red-shifted emission is undesirable for applications requiring short-wavelength or high-color-purity chromophores. Therefore, in this study, para-linked configurations at positions 2 and 5 were intentionally excluded to prioritize a focused investigation of ortho- (position 7) and meta-linked (position 4) derivatives [16]. In the case of ortho- and meta-substitution, the molecules adopt a highly twisted conformation in order to achieve structural stability, which results in a relatively greater angle strain compared to the para position [17]. Consequently, the dihedral angle increases to nearly 90°, leading to reduced π-orbital overlap and preventing the extension of conjugation. Therefore, it is expected that this structural feature can be utilized to tune the emission wavelength toward the blue region. This approach enables the fine-tuning of electronic communication through spatial arrangement, as previously reported [18]. The synthetic route is illustrated in Scheme 1, and detailed procedures are provided in Section 2: Synthesis and Characterization. The synthesis of the target compounds involved a Buchwald–Hartwig cross-coupling reaction and a palladium-catalyzed intramolecular cyclization, as previously reported [19,20,21]. In the final step, a self-coupling reaction occurred to form the dimeric structure, which was confirmed by both NMR spectroscopy and mass spectrometry (Figures S1–S4). These results clearly verified the successful formation of the desired dimer [22,23,24].

### 3.2. Photophysical Properties

**Figure 2:**
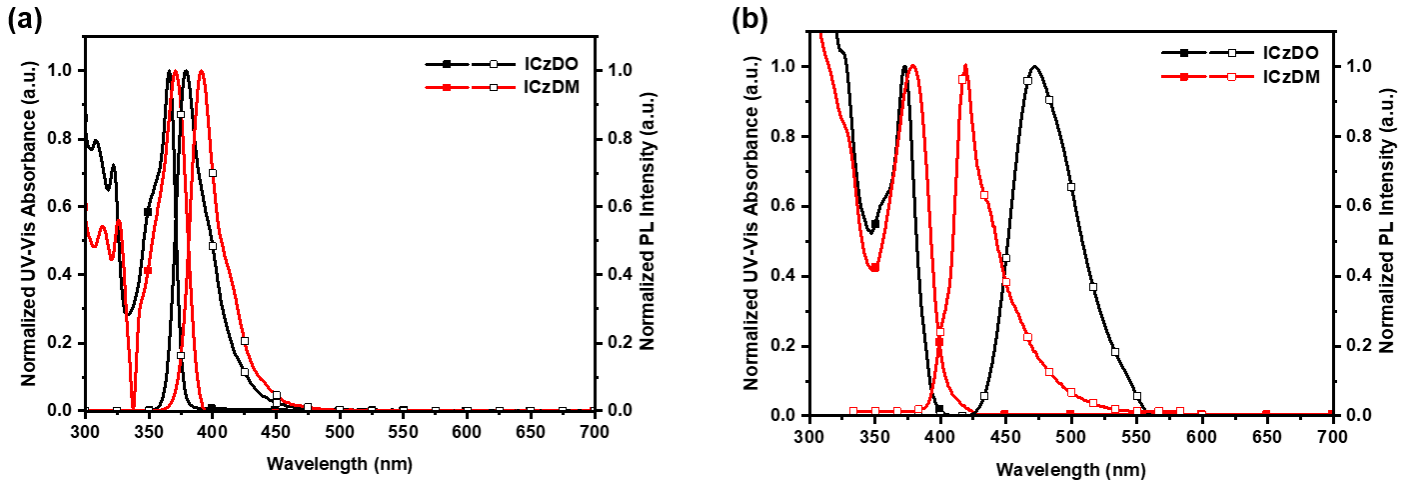
Normalized UV–Vis absorption and PL spectra of the synthesized compounds measured in (**a**) dilute toluene solution (1 × 10^−5^ M) and (**b**) vacuum-deposited thin films (thickness: 50 nm).

The optical properties of the newly synthesized compounds ICzDO and ICzDM were characterized by measuring their ultraviolet-visible (UV-Vis) absorption and PL spectra in both toluene solutions and vacuum-deposited thin-film states. The results are presented in Figure 2 and tabulated in Table 1. In solution, both ICzDO and ICzDM exhibited absorption in the range of 300–400 nm, with strong absorption bands observed between 360 and 380 nm. These peaks originate from π–π* electronic transitions, which are localized due to the highly planar and rigid structure of the ICz backbone. The maximum absorption wavelengths (λ_abs_) were found to be 366 nm for ICzDO and 371 nm for ICzDM [25].

HOMO energy levels were evaluated using ultraviolet photoelectron spectroscopy (UPS, Riken–Keiki, AC-2). The optical band gaps were estimated from the onset of absorption, derived from plots of (*hν*) versus (*αhν*)^2^, where α is the absorption coefficient, h is Planck’s constant, and ν is the frequency of incident light. The LUMO energy levels were calculated by subtracting the optically derived band gaps from the experimentally determined HOMO levels. As shown in Table 1, ICzDO exhibited a HOMO level of −5.70 eV, with a band gap of 3.43 eV, resulting in a LUMO level of −2.27 eV. Similarly, ICzDM had a HOMO energy of −5.78 eV and a band gap of 3.08 eV, yielding a LUMO value of −2.70 eV. The HOMO energy levels of ICzDO and ICzDM were found to be nearly identical, which can be attributed to the delocalization of electron density across the entire ICz core. In contrast, the optical band gap of ICzDO was 0.35 eV larger than that of ICzDM. This difference is likely due to the larger dihedral angle in ICzDO, which reduces effective conjugation between the linked units [26].

ICzDO and ICzDM exhibited peak PL emissions at 379 nm and 391 nm. Their emission bandwidths were remarkably narrow, with FWHM values of 28 nm (ICzDO) and 26 nm (ICzDM), both under 30 nm. The Stokes shifts, determined from the difference between the UV absorption and PL maxima, were found to be 13 nm for ICzDO and 20 nm for ICzDM, reflecting the rigid molecular structures and suppressed vibrational relaxation of both materials. The narrow emission bandwidths and minimal Stokes shifts observed for ICzDO and ICzDM can be attributed to the rigid nature of the ICz framework, which limits molecular motions such as vibration and rotation [7]. In comparison, the para-linked analog at the 2-position, 2,2′-biindolo[3,2,1-jk]carbazole (ICzDP), was reported to emit at 406 nm under identical conditions (5 μM in dichloromethane), whereas ICzDO and ICzDM exhibited blue-shifted PL maxima. These findings highlight the substantial effect of the linkage position on the optical properties of ICz-based chromophores [27].

Photoluminescence measurements in the thin-film state revealed λ_p1_ values of 507 nm for ICzDO and 428 nm for ICzDM. When compared to their respective solution-state emissions, ICzDO showed a significantly larger shift of 128 nm, while ICzDM exhibited a red-shift of 37 nm [28]. These shifts can be attributed to closer molecular packing in the solid state, which promotes intermolecular interactions and results in red-shifted emission accompanied by spectral broadening. In the case of ICzDM, the observed film-state emission properties followed conventional trends. However, ICzDO showed an exceptionally large red shift exceeding 100 nm, together with a broad emission band characterized by an FWHM of 87 nm. Such spectral features are indicative of excimer emission. To verify whether the emission at 507 nm arises from excimer formation, the excitation spectrum of ICzDO in the solid state was measured at room temperature (RT) (Figure S5). Excitation spectra measured at the PL maxima of ICzDO in solution (379 nm) and in the film state (507 nm) revealed a high degree of similarity, with both closely matching the absorption spectrum. This observation indicates that both emissions originate from the same excited state pathway [29]. These findings support the conclusion that ICzDO exhibits excimer emission in the film state.

**Figure 3:**
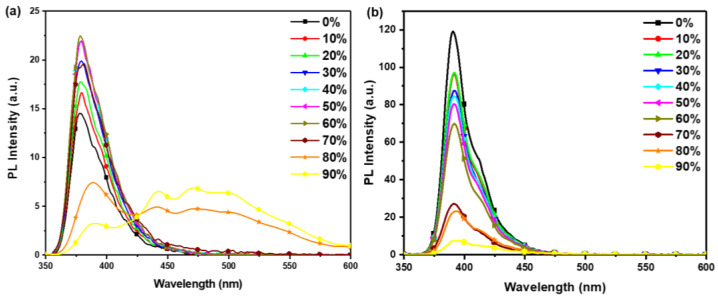
Variation in PL intensity of (**a**) ICzDO and (**b**) ICzDM with increasing water fraction in THF/water mixtures.

**Figure 4:**
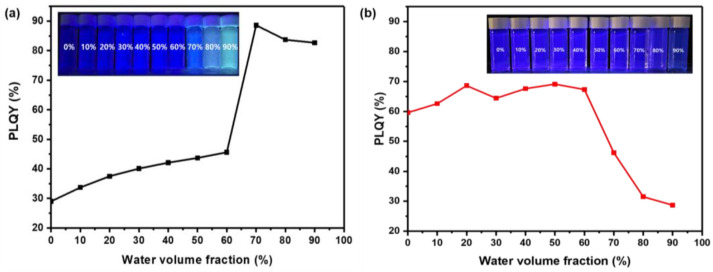
Variation in PLQY with increasing water content for (**a**) ICzDO and (**b**) ICzDM in THF/water mixtures.

PLQYs of ICzDO and ICzDM in solution were measured to be 26.2% and 48.7%, respectively. In contrast, their PLQYs in the film state were 80.0% for ICzDO and 35.3% for ICzDM. To further investigate the unexpectedly high PLQY of ICzDO in the solid state, a water fraction experiment using THF/H_2_O mixtures was carried out to confirm the presence of AEEE (Figure 3). ICzDO was prepared in THF at a concentration of 10^−5^ M, and its PL behavior was monitored by gradually increasing the H_2_O fraction from 0% to 90%. In the range of 0–70% H_2_O, the PL intensity increased in the 350–425 nm region, accompanied by bright blue emission. However, the PL intensity decreased at H_2_O fractions above 80%, and the emission spectrum became significantly broader, extending across the 350–600 nm range. As the H_2_O fraction increased, ICzDO exhibited typical AIE behavior, attributed to reduced intermolecular distances. The strong intermolecular interactions led to the formation of excimers [14,30,31]. The PLQY gradually increased with H_2_O content up to 60%, and a sharp enhancement was observed at 70%, where the PLQY reached 88.6% (Figure 4). Even at higher water fractions (80–90%), ICzDO maintained high PLQY values above 80%, indicating efficient solid-state emission. In contrast, ICzDM exhibited a typical ACQ effect, as both PL intensity and PLQY decreased with increasing H_2_O fraction. As shown in Figure 3b, the PL intensity of ICzDM exhibited a progressive decline as the H_2_O fraction increased from 0% to 60%. However, a sharp reduction in PL spectral intensity was observed at H_2_O concentrations exceeding 70%, likely due to aggregation-induced quenching effects in the aqueous environment. While PLQY showed minimal reduction (0% to 60% H_2_O) compared to the pronounced PL intensity decrease, a sharp PLQY decline occurred at over 70% H_2_O. This contrast can be attributed to PL intensity’s susceptibility to experimental variables (e.g., light scattering, concentration effects), in contrast to PLQY’s intrinsic stability as a normalized parameter that inherently compensates for external optical artifacts. Furthermore, its emission maximum remained unchanged at 391 nm, regardless of the water content. These observations suggest that ICzDO, unlike ICzDM, demonstrates AEEE, resulting in high PLQY in the solid state and excimer emission at 507 nm. The presence of AEEE behavior in ICzDO implies that it is likely to retain favorable emission characteristics when applied in OLED devices.

**Figure 5:**
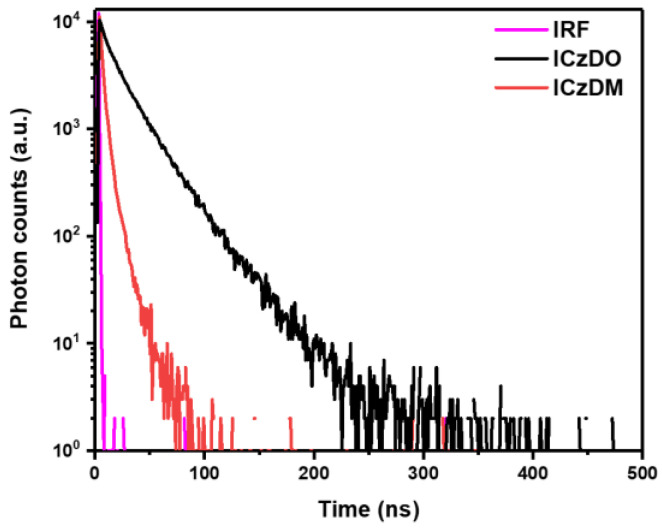
PL lifetime measurements of the evaporated films (thickness: 50 nm) obtained from time-resolved photoluminescence decay (IRF included. Time window—1 ns).

To provide additional support for the occurrence of AEEE in the solid state, transient photoluminescence (TRPL) measurements were carried out. First, in the solution state, the monomer ICz exhibited comparable PLQY and decay times to ICzDO (Figure S6 and Table S1). ICzDM, however, demonstrated a higher PLQY and a faster radiative decay rate (*k_rad_* = 13.4 × 10^7^ s^−1^) compared to both materials. Since excited-state dynamics in thin-film states are critical for evaluating optoelectronic performance, the photophysical properties of ICzDO and ICzDM were further investigated in evaporated films with a thickness of 50 nm (Figure 5). ICzDO exhibited a fluorescence lifetime (τ_F_) of 23.3 ns, whereas ICzDM showed a much shorter fluorescence lifetime (τ_F_) of 4.13 ns, which is characteristic of typical prompt fluorescence. The extended lifetime observed for ICzDO supports the presence of excimer emission contributing to its AEEE characteristics. The longer τ_F_ observed for ICzDO, compared to ICzDM, can be attributed to the additional time required for energy transfer involved in excimer emission. This delay is characteristic of AEEE-type processes [32]. In thin films, ICzDO showed the radiative rate constant (*k_rad_*) of 3.43 × 10^7^ s^−1^, whereas ICzDM exhibited a higher value of 8.58 × 10^7^ s^−1^. Meanwhile, the non-radiative rate constant (*k_nr_*) values were 0.86 × 10^7^ s^−1^ for ICzDO and 15.6 × 10^7^ s^−1^ for ICzDM, suggesting that ICzDO has significantly reduced non-radiative decay, contributing to its high PL efficiency. While ICzDM showed a higher *k_rad_*, its *k_nr_* was also substantially higher. Consequently, the *k_rad_/k_nr_* ratio was greater for ICzDO (4.00) than for ICzDM (0.54). This suggests that the enhanced PLQY observed for ICzDO in the solid state is primarily due to its more favorable balance between radiative and non-radiative decay pathways. To investigate thickness-dependent properties in more detail, thin films were additionally fabricated via vacuum deposition at 10 nm thickness and compared with those of 50 nm thickness (Figure S7 and Table S2). PLQY in thin films can be influenced by multiple factors, including substrate-induced coherent effects, self-reabsorption, energy transfer between molecules, aggregation states, and out-coupling efficiency variations with film thickness. Further detailed studies on these contributing factors will be conducted in future work. PLQY measurements revealed that ICzDM exhibited increased emission efficiency with thicker films, whereas ICzDO showed a slight PLQY reduction (81.9% at 10 nm vs. 80.0% at 50 nm), likely due to excimer formation. Contrary to expectations, the decay time of ICzDO decreased in the 50 nm film despite the higher excimer population, which typically correlates with prolonged decay kinetics. This suggests that non-radiative decay pathways may dominate in thicker ICzDO films, counteracting the anticipated excimer-induced lifetime extension. This phenomenon is likely attributable to an accelerated *k_nr_* in thicker films, which reduces the overall decay time, as reported in prior studies [33]. For ICzDM, while thicker films enhanced PLQY, the observed elongation of decay time and reduction in *k_rad_* suggest that strong intermolecular interactions dominate the excited-state dynamics at increased film thicknesses.

### 3.3. Theoretical Calculation

**Figure 6:**
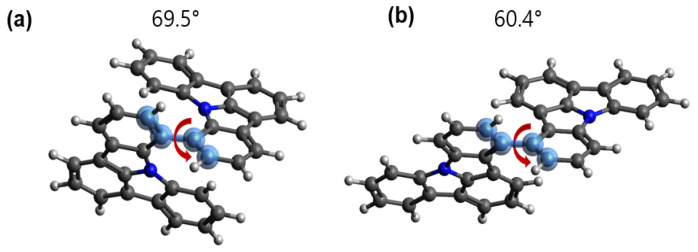
DFT-optimized structures and corresponding dihedral angles of (**a**) ICzDO and (**b**) ICzDM.

**Figure 7:**
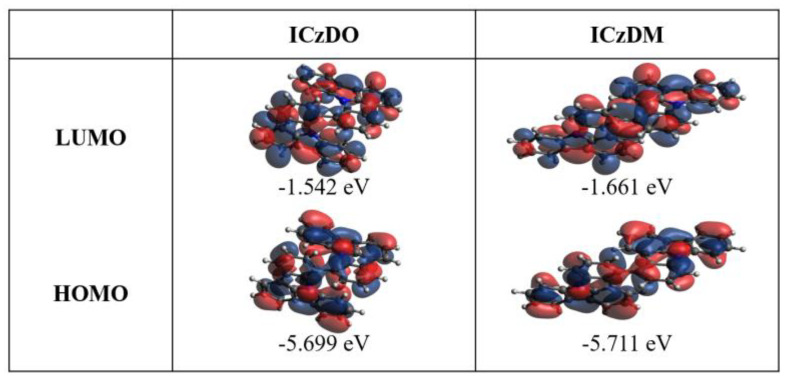
HOMO and LUMO electron density distributions of ICzDO and ICzDM based on B3LYP/6-311G(d,p) calculations.

Molecular structure optimization was carried out using the ORCA program at the B3LYP-D3/def2-TZVPP level to further investigate the conformational characteristics of the compounds. The calculated dihedral angles between the two ICz chromophores were 69.5° for ICzDO and 60.4° for ICzDM, indicating a moderately twisted geometry in both cases. Although the angles are comparable, ICzDO exhibited a slightly greater twist (Figure 6). Such dihedral-induced strain may contribute to the blue-shifted emission observed in solution, and this behavior aligns well with the optical characteristics previously reported. According to the molecular orbital calculations (Figure 7), the HOMO, LUMO, and band gap values were found to be −5.69, −1.54, and 4.15 eV for ICzDO, and −5.71, −1.66, and 4.05 eV for ICzDM. These values are in good agreement with the experimentally determined trends (Figure 7).

### 3.4. Thermal Properties and Morphology

**Figure 8:**
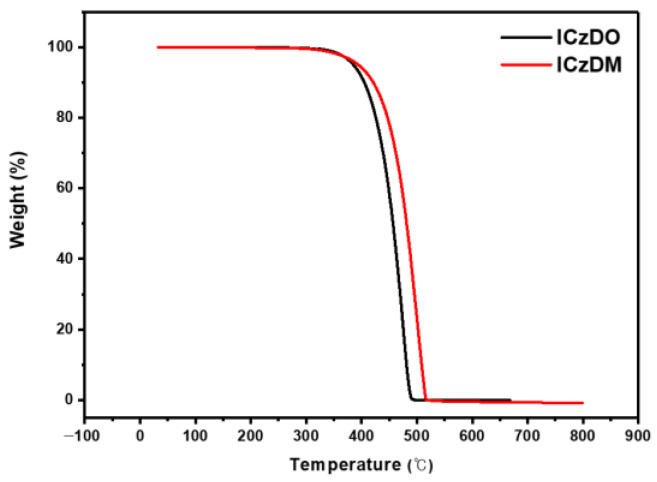
TGA profiles of ICzDO and ICzDM measured with a heating rate of 10 °C/min.

**Figure 9:**
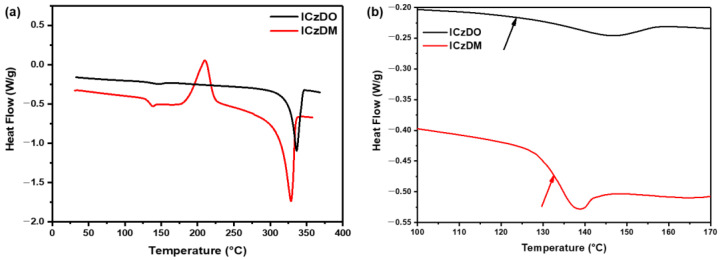
Thermal properties of ICzDO and ICzDM measured at a heating rate of 10 °C/min: (**a**) DSC curves of ICzDO and ICzDM; (**b**) enlarged view of the glass transition regions (T_g_) for ICzDO and ICzDM.

As illustrated in Figure 8 and Figure 9, the thermal properties of ICzDO and ICzDM were investigated via TGA and DSC. The decomposition temperatures (T_d_, 5% weight loss) determined from TGA were 386 °C for ICzDO and 390 °C for ICzDM, suggesting similar thermal decomposition behavior. These elevated T_d_ values exceed the minimum temperature requirement of 200 °C for OLED fabrication processes, indicating that the materials possess sufficient thermal stability for device integration. Furthermore, such high thermal robustness is expected to enhance device longevity by improving resistance to joule heating generated during OLED operation. The T_g_ values were found to be 123.6 °C and 132.5 °C, while T_c_ values were 290 °C and 210 °C, and T_m_ values were 336 °C and 328 °C for ICzDO and ICzDM, respectively. The slightly lower T_m_ of ICzDM can be attributed to weaker intermolecular interactions, in contrast to ICzDO, which exhibits strong interactions likely due to excimer formation [34]. To investigate the morphology of ICzDO and ICzDM, atomic force microscopy (AFM) measurements were performed on thin films fabricated by vacuum deposition. The measured root mean square (RMS) roughness values were 1.74 nm for ICzDO and 1.33 nm for ICzDM, demonstrating that both films possess comparably smooth surfaces. Such smooth thin-film characteristics are expected to facilitate efficient charge transfer between layers during device fabrication (Figure S8).

### 3.5. Electroluminescence Properties

**Figure 10:**
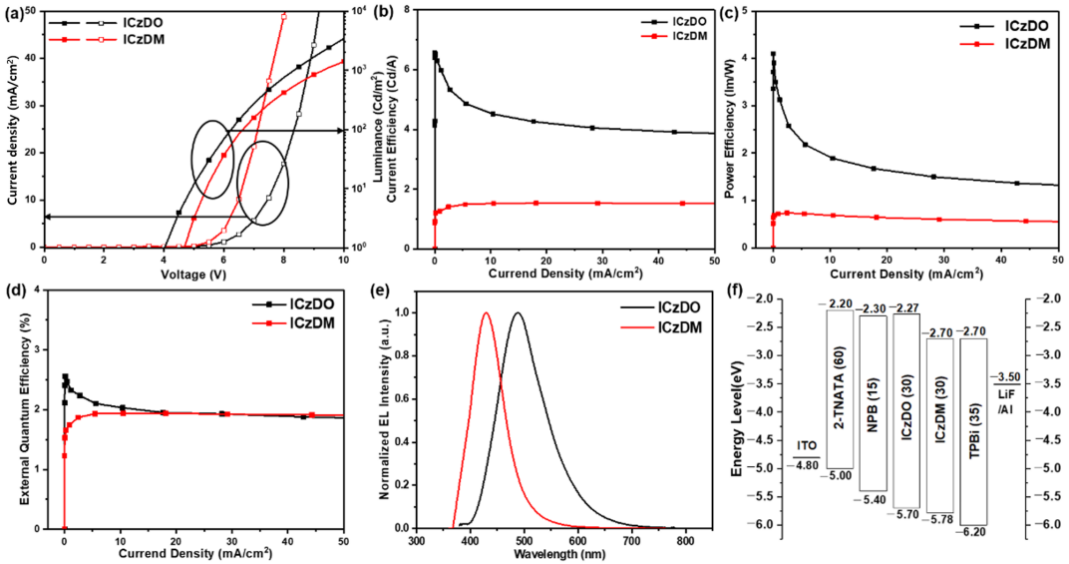
EL performance of non-doped devices using ICzDO and ICzDM as the EML: (**a**) J–V–L characteristics; (**b**) CE vs. J curves; (**c**) PE vs. J curves; (**d**) EQE vs. J curves; (**e**) EL spectra of the non-doped devices; and (**f**) band diagrams of the fabricated OLED devices. Device configuration: ITO/2TNATA (60 nm)/NPB (15 nm)/EML (30 nm)/TPBi (35 nm)/LiF (1 nm)/Al (200 nm).

To evaluate the electroluminescent performance of ICzDO and ICzDM, non-doped OLED devices were developed, with these compounds serving as the emitting layers (EML). The device configuration, depicted in Figure 10 and summarized in Table 2, consisted of the following structure: ITO/2-TNATA (60 nm)/NPB (15 nm)/ICzDO or ICzDM (30 nm)/TPBI (35 nm)/LiF (1 nm)/Al (200 nm). This structure was used to directly evaluate the intrinsic emission properties of the materials without any dopants. The device used 2-TNATA as the hole injection layer (HIL) and NPB as the hole-transporting layer (HTL) to facilitate hole transport, while TPBI acted as the electron transport layer (ETL) and LiF was applied to promote electron injection as the electron injection layer (EIL). ITO and Al were used as the anode and cathode materials. The chemical structures of the materials used for device fabrication are shown in Figures S9 and S10. Figure 10 provides the energy level diagram and chemical structures of the materials incorporated in the non-doped OLED devices. The non-doped OLED devices demonstrated typical J–V–L behavior. Under a current density of 10 mA/cm^2^, ICzDO operated at 7.50 V, while ICzDM required only 6.50 V. This 1.0 V difference is likely due to the relatively higher LUMO level of ICzDO, which poses a greater barrier to electron injection compared to ICzDM. The current efficiency (CE) of the ICzDO-based device was 4.51 cd/A, which is three times higher than that of ICzDM (1.5 cd/A). In addition, the external quantum efficiency (EQE) of ICzDO was slightly higher at 2.09%, compared to 1.93% for ICzDM. This enhanced performance is attributed to the high PLQY observed in the film state, as previously discussed, originating from the AEEE behavior of ICzDO. The EL characteristics indicate that ICzDO exhibited an EL_max_ at 492 nm and CIE coordinates of (0.209, 0.332), representing a red-shifted emission relative to ICzDM, which emitted at 429 nm with CIE coordinates of (0.153, 0.092). These EL maxima were consistent with the film-state PL wavelengths. As a result, ICzDM successfully achieved bright emission in the deep-blue region, with color coordinates approaching the National Television System Committee (NTSC) television standard for blue.

**Figure 11:**
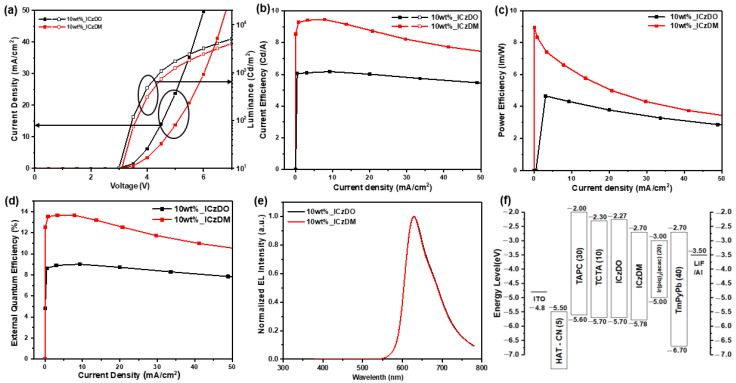
EL performance of doped devices using ICzDO and ICzDM as the EML: (**a**) J–V–L characteristics; (**b**) CE vs. J curves; (**c**) PE vs. J curves; (**d**) EQE vs. J curves; (**e**) EL spectra of the doped devices at 10 mA/cm^2^; and (**f**) band diagrams of the fabricated OLED devices. Device configuration: ITO/HAT-CN (5 nm)/TAPC (30 nm)/TCTA (10 nm)/Host: 10 wt% Ir(piq)_2_(acac) (20 nm)/LiF (1 nm)/Al (200 nm).

To further explore their potential as host materials, ICzDO and ICzDM, featuring high photoluminescence efficiency and elevated triplet energy levels (T_1_), were applied in doped OLED devices designed for red phosphorescent emission. The performance characteristics of these devices are presented in Figure 11 and Table 3. The triplet energies of ICzDO (2.79 eV) and ICzDM (2.72 eV) exceed that of the phosphorescent dopant Ir(piq)_2_(acac) (1.98 eV), ensuring favorable conditions for Dexter-type energy transfer from host to dopant (Figure S11) [35]. Red doped OLED devices were fabricated using the synthesized compounds as host matrices. The device structure consisted of the following: ITO/1,4,5,8,9,11-hexaazatriphenylenehexacarbonitrile (HAT-CN, 5 nm)/TAPC (30 nm)/TCTA (10 nm)/host blended with 10 wt% Ir(piq)_2_(acac) (20 nm)/TmPyPb (40 nm)/LiF (1 nm)/Al (200 nm). This multilayer stack was optimized to support efficient exciton formation and emission in the red spectral region. In this device configuration, HAT-CN functioned as the HIL, TAPC was used for hole transport, and TCTA served as the exciton blocking layer (EBL). LiF and Al acted as the EIL and cathode, while TmPyPb operated as both ETL and hole blocking layer (HBL). At a current density of 10 mA/cm^2^, the device using ICzDO as the host showed acceptable performance, with a CE of 6.17 cd/A and an EQE of 9.00%. In contrast, the device incorporating ICzDM as the host achieved a significantly higher CE_max_ of 9.34 cd/A and EQE_max_ of 13.5%. The non-doped device results were consistent with the photophysical characteristics observed in the film state, where ICzDO, having a higher PLQY, delivered better EQE [36]. In contrast, ICzDM achieved higher EQE in the doped devices. To explain this result, the degree of spectral overlap between the PL spectra of the host materials (film state) and the UV absorption spectrum of Ir(piq)_2_(acac) (solution state) was examined, as shown in Figure S12. Evaluating the overlap between the UV absorption spectrum of the dopant and the PL emission spectrum of the host allows for a qualitative comparison of energy transfer effectiveness. Figure S12 illustrates that Ir(piq)_2_(acac) absorbs broadly from 300 to 600 nm. It is evident that the film-state PL spectrum of ICzDM aligns more significantly with this absorption range than that of ICzDO, indicating a stronger spectral match and more efficient energy transfer potential for ICzDM [37]. The superior device performance of ICzDM is due to more effective energy transfer to Ir(piq)_2_(acac), in comparison to ICzDO. As shown by the EL characteristics, both devices exhibited red emission peaks at 628 nm (ICzDM) and 629 nm (ICzDO), with CIE coordinates of (0.684, 0.315) and (0.685, 0.314), respectively. These values confirm the high red color purity of the emission and imply that a stable recombination zone was successfully formed in the EML where the dopant is present, ensuring efficient radiative recombination.

## 4. Conclusions

This work demonstrates the development and evaluation of new ICz-based red host materials for phosphorescent OLEDs. By constructing dimer-type structures, we revealed that the position of molecular linkage plays a crucial role in determining the overall properties of the compounds. The findings highlight that proper control of the linking position not only induces AEEE characteristics but also contributes to achieving highly efficient red doped devices. The inherent rigidity of ICzDO and ICzDM led to sharp, short-wavelength PL emissions with narrow FWHM in solution. In contrast, film-state studies revealed that ICzDM was prone to ACQ effects, whereas ICzDO showed enhanced PL performance due to AEEE-induced excimer formation, resulting in a notable increase in PLQY. In the non-doped OLED devices, ICzDO demonstrated a slightly higher EQE of 2.09% compared to 1.93% for ICzDM. However, in the red phosphorescent doped devices using Ir(piq)_2_(acac) as the dopant, ICzDM achieved a significantly higher EQE of 13.5%, while ICzDO showed 9.00%. This performance enhancement is attributed to the more efficient energy transfer in the ICzDM-based device, resulting in both superior EQE and excellent red color purity. The superior EQE performance of ICzDM highlights a key insight that modifying the linkage position of an identical chromophore unit can lead to remarkable improvements in OLED characteristics. In conclusion, this work establishes the potential of designing dimer-type molecules with positional variation as a strategic approach to realizing high-efficiency OLEDs with excellent color purity.

## Figures and Tables

**Table 1:** Summary of photophysical and electrical characteristics of the synthesized compounds.

	Solution ^a^			Film ^b^									
	λ_abs_ (nm)	λ_PL_ (nm)	FWHM (nm)	λ_abs_ (nm)	λ_PL_ (nm)	FWHM (nm)	ф_PL_ ^c^ (%)	τ_F_ ^d^ (ns)	*k_rad_* ^e^ (10^7^/s)	*k_nr_* ^e^ (10^7^/s)	HOMO ^f^ (eV)	LUMO (eV)	Band Gap (eV)
ICzDO	366	379	28	372	507	87	26.2/80.0	23.3	3.43	0.86	−5.70	−2.27	3.43
ICzDM	371	391	26	379	428	50	48.7/35.3	4.13	8.58	15.6	−5.78	−2.70	3.08

^a^ Toluene, 10^−5^ M; ^b^ film thickness is 50 nm; ^c^ absolute photoluminescence quantum yield of solution state/film state; ^d^ fluorescence life time in film state; ^e^ values of *k_rad_* and *k_nr_* were calculated according to the equations of *k_rad_* = Φ_F_/τ_F_ and *k_nr_* = (1/τ_F_) − *k_rad_*; ^f^ ultraviolet photoelectron spectroscopy (Riken-Keiki, AC-2).

**Table 2:** Comparison of the EL performance of the non-doped OLED devices measured at 10 mA/cm^2^.

	Voltage (V)	CE (cd/A)	PE (lm/W)	EQE (%)	CIE (x,y)	EL_max_ (nm)	FWHM (nm)
ICzDO	7.50	4.51	1.89	2.09	(0.209, 0.332)	492	100
ICzDM	6.50	1.51	0.68	1.93	(0.153, 0.092)	429	70

**Table 3:** Comparison of the EL performance of the doped OLED devices measured at 10 mA/cm^2^.

	Voltage (V)	CE (cd/A)	PE (lm/W)	EQE (%)	CIE (x,y)	EL_max_ (nm)	FWHM (nm)
ICzDO	4.25	6.17	4.28	9.00	(0.685, 0.314)	629	84
ICzDM	4.70	9.34	6.29	13.5	(0.684, 0.315)	628	84

## Data Availability

The original contributions presented in this study are included in the article and Supplementary Materials. Further inquiries can be directed to the corresponding author.

## Supplementary Materials

The following supporting information can be downloaded at: https://www.mdpi.com/article/10.3390/ma18092058/s1, Figure S1: ^1^H NMR spectrum of ICzDO (7,7′-biindolo[3,2,1-jk]carbazole); Figure S2: ^1^H NMR spectrum of ICzDM (4,4′-biindolo[3,2,1-jk]carbazole); Figure S3: GC-HRMS spectrum of ICzDO (7,7′-biindolo[3,2,1-jk]carbazole); Figure S4: GC-HRMS spectrum of ICzDM (4,4′-biindolo[3,2,1-jk]carbazole); Figure S5: UV absorption and excitation spectra of the ICzDO film (thickness: 50 nm): UV absorption spectrum; excitation spectrum monitored at 379 nm; and excitation spectrum monitored at 507 nm; Figure S6: PL lifetime measurements in solution state (IRF included. Time window: 1 ns); Figure S7: TRPL curves depending on the film thickness of the synthesized materials; Figure S8: AFM images (a) ICzDO and (b) ICzDM; Figure S9: Chemical structures of the materials used in the non-doped devices; Figure S10: Chemical structures of the materials used in the doped devices; Figure S11: PL spectra of ICzDO and ICzDM measured at room temperature (RT) and low temperature (LT); Figure S12: Overlap of the emission spectrum of Ir(piq)_2_(acac) with the absorption spectra of the newly synthesized materials; Table S1: Photophysical properties of ICz and the synthesized materials in solution state; Table S2: Thickness-dependent photophysical properties of evaporated thin films of the synthesized materials.
