# Supplementary material for: Electrical and Optical Properties Depending on the Substitution Position of a Novel Indolocarbazole Dimer
Source: Materials (Basel). 2025 Apr 30;18(9):2058. doi: 10.3390/ma18092058 (PMC12072749; doi:10.3390/ma18092058)
Supplement: Supplementary file 1 [file materials-18-02058-s001.zip › materials-3592152-supplementary.pdf]

Electrical and Optical Properties Depending on the Substitution Position of a Novel Indolocarbazole Dimer

Jiyun Kim[1, †], Suhyeon Jeong[1, †], Sangwook Park[1], Saeyoung Oh[1], Soonhang Lee[2], Jihoon Lee[2], Hayoon Lee[1], and Jongwook Park[1, *]

[1]*Integrated Engineering, Department of Chemical Engineering, Kyung Hee University, Yongin, 17104, Republic of Korea*

[2]*Department of Polymer Science and Engineering and Department of IT·Energy Convergence (BK21 FOUR), Korea National University of Transportation, Chungju 27469, Republic of Korea*

Corresponding authors. E-mail addresses: Jongpark@khu.ac.kr (J. Park)

†Jiyun Kim and Suhyeon Jeong contributed equally to this work as the first coauthor.

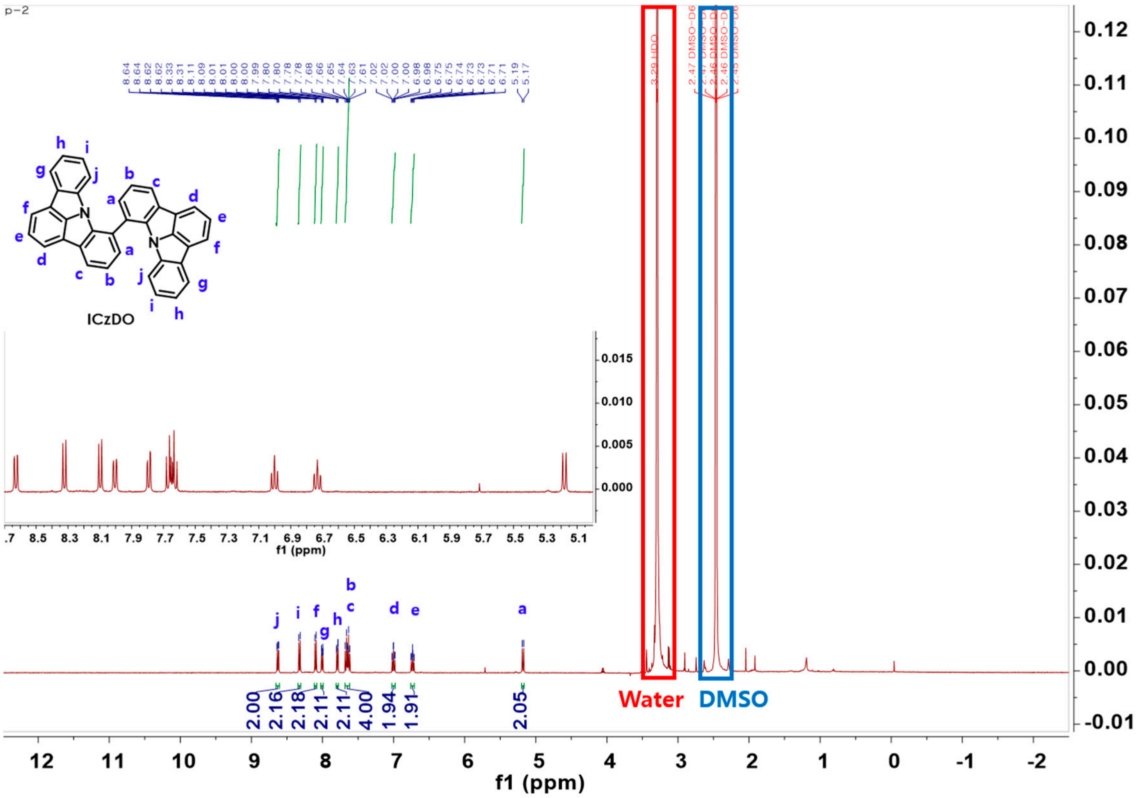

Figure S1. ^{1}H NMR spectrum of ICzDO (7,7'-biindolo[3,2,1-jk]carbazole).

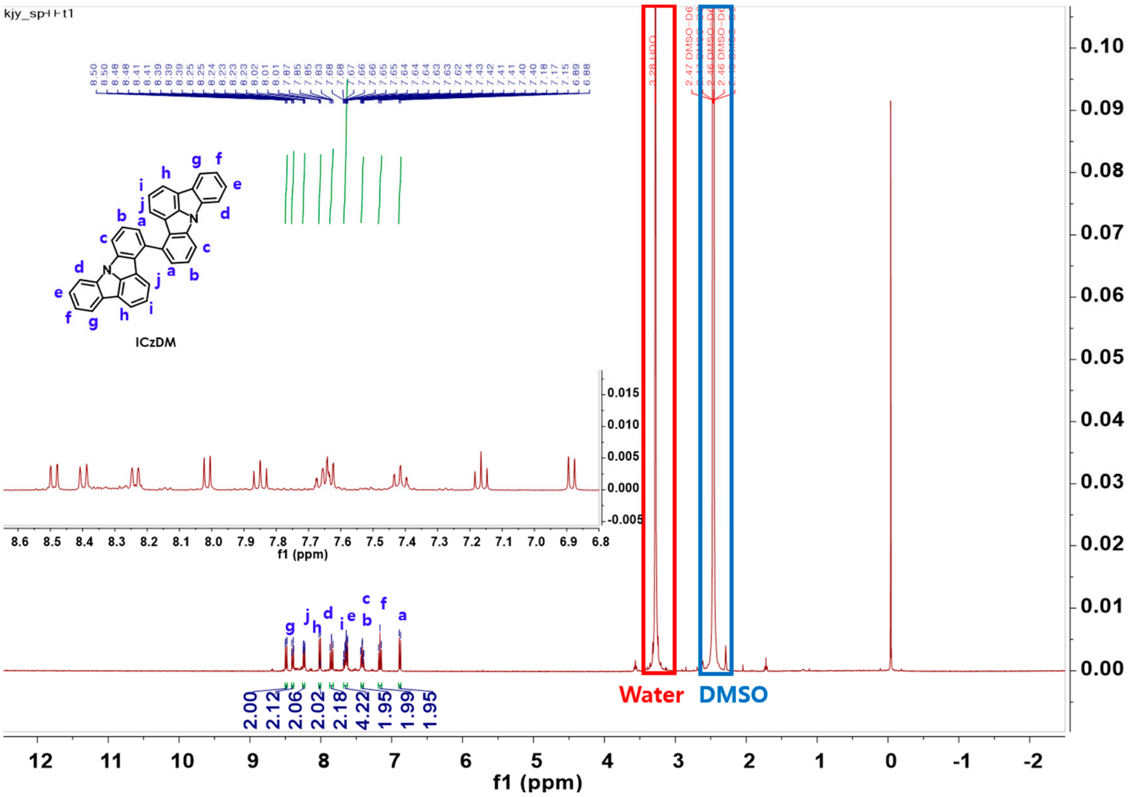

Figure S2. ^{1}H NMR spectrum of ICzDM (4,4'-biindolo[3,2,1-jk]carbazole).

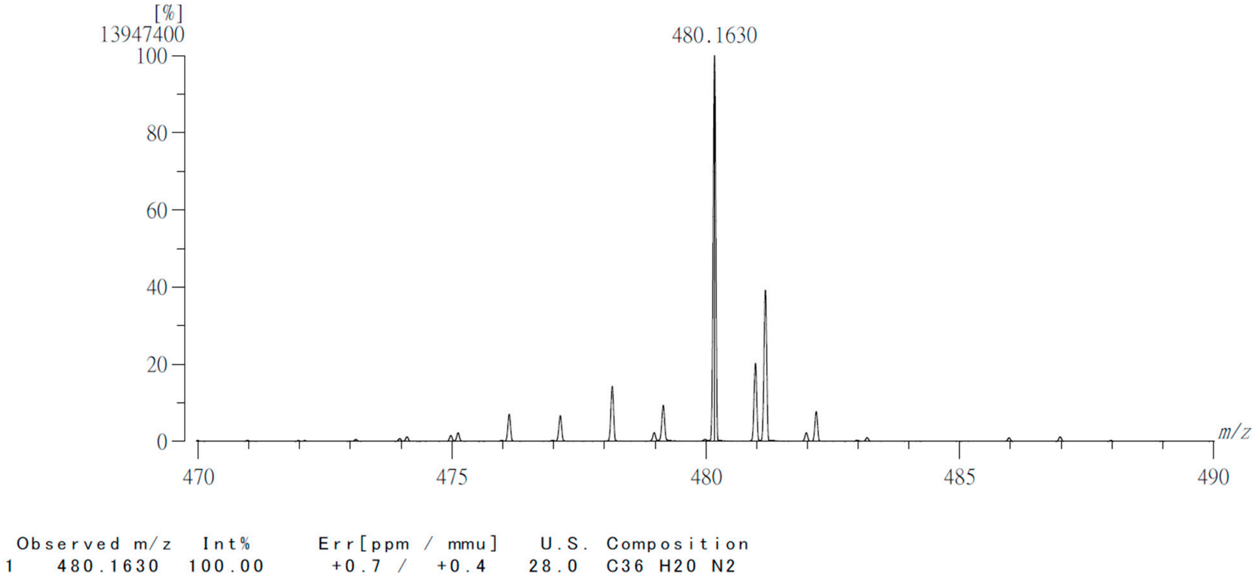

Figure S3. GC-HRMS spectrum of ICzDO (7,7'-biindolo[3,2,1-jk]carbazole).

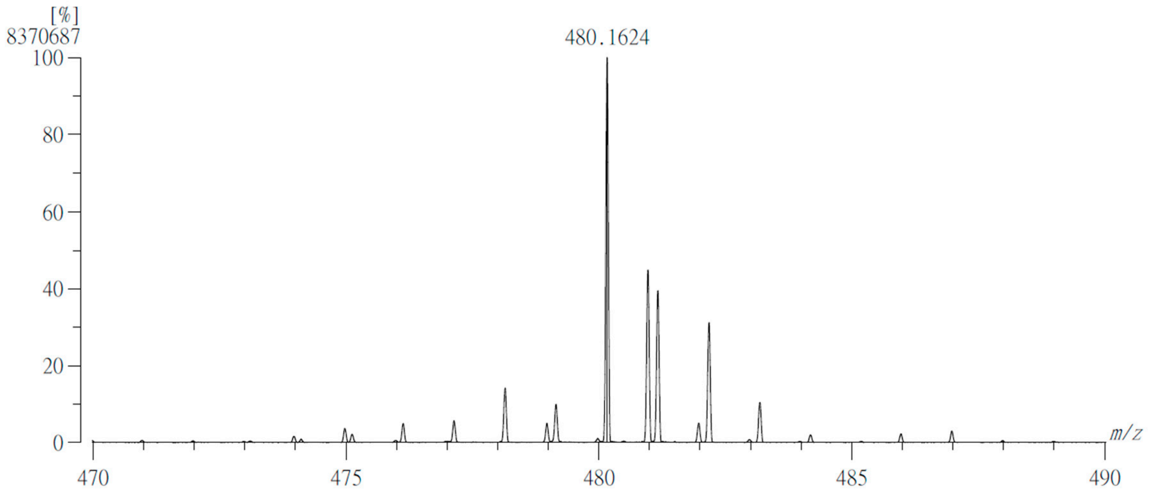

	Observed m/z	Int%	Err[ppm / mmu]	U.S.	Composition
1	480.1624	100.00	-0.5 / -0.2	28.0	C36 H20 N2

Figure S4. GC-HRMS spectrum of ICzDM (4,4'-biindolo[3,2,1-jk]carbazole).

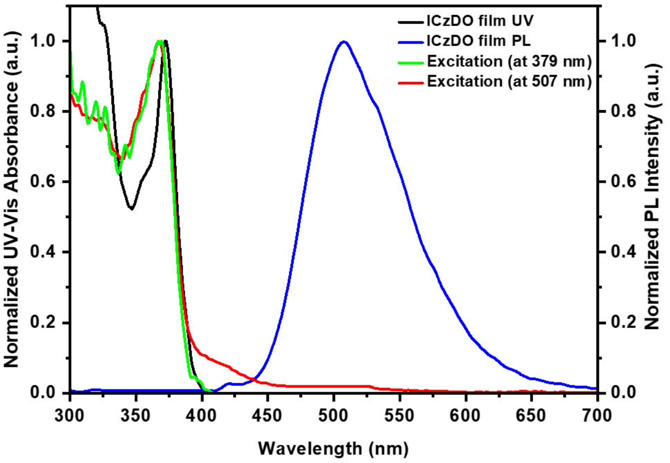

Figure S5. UV absorption and excitation spectra of the ICzDO film (thickness: 50 nm): UV absorption spectrum; excitation spectrum monitored at 379 nm; and excitation spectrum monitored at 507 nm.

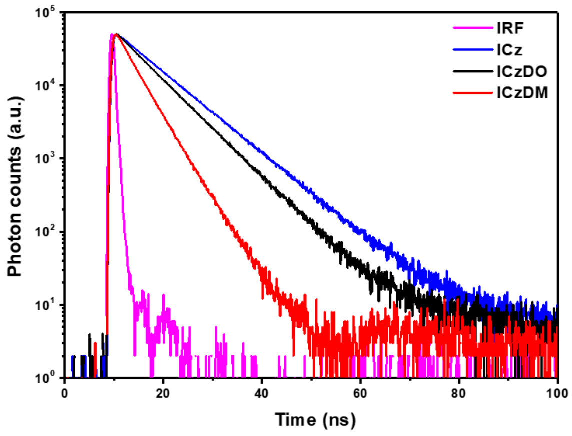

Figure S6. PL lifetime measurements in solution state (IRF included, time window: 1 ns).

Table S1. Photophysical properties of ICz and the synthesized materials in solution state.

	Φ_{PL} (%)	τ_F (ns)	k_{rad} (10^7/s)	k_{nr} (10^7/s)
**ICz**	**28.7**	**7.78**	**3.69**	**9.16**
**ICzDO**	**26.2**	**6.49**	**4.04**	**11.4**
**ICzDM**	**48.7**	**3.64**	**13.4**	**14.1**

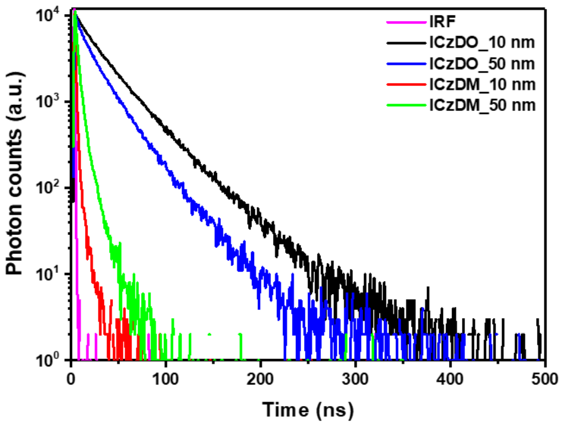

Figure S7. TRPL curves depending on the film thickness of the synthesized materials.

Table S2. Thickness-dependent photophysical properties of evaporated thin films of the synthesized materials.

	ϕ_{PL} (%)	τ_F (ns)	k_{rad} (10^7/s)	k_{nr} (10^7/s)
ICzDO_10 nm	81.9	32.6	2.51	0.56
ICzDO_50 nm	80.0	23.3	3.43	0.86
ICzDM_10 nm	23.1	1.22	18.9	63.1
ICzDM_50 nm	35.3	4.13	8.58	15.7

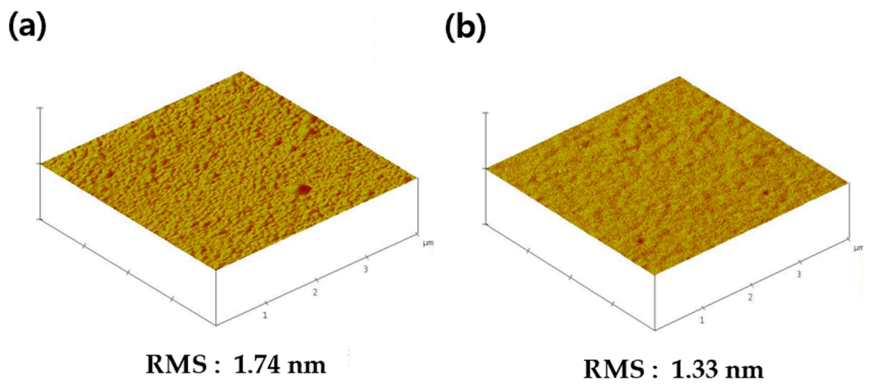

Figure S8. AFM images (a) ICzDO and (b) ICzDM.

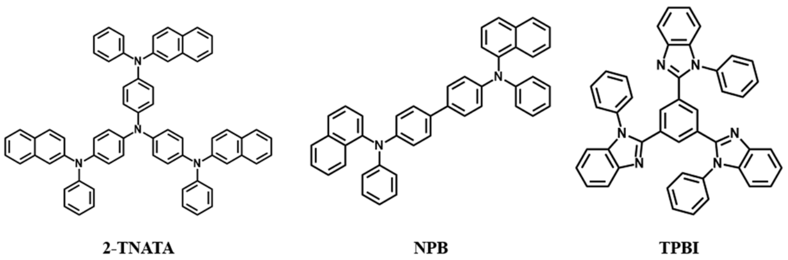

Figure S9. Chemical structures of the materials used in the non-doped devices.

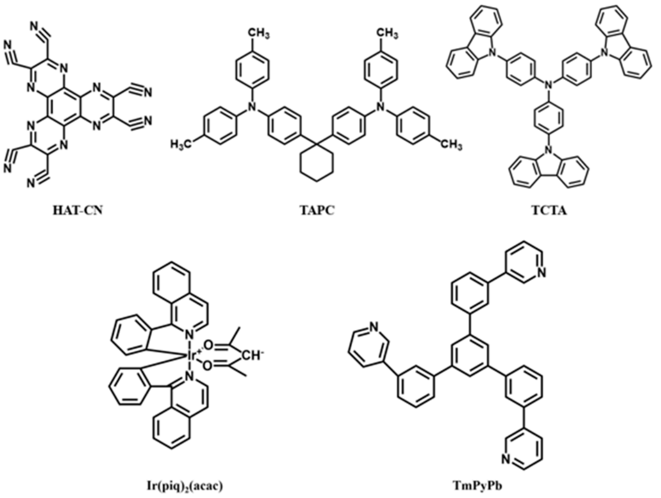

Figure S10. Chemical structures of the materials used in the doped devices.

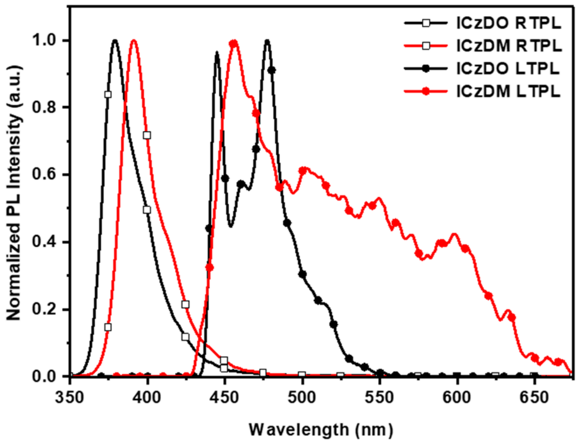

Figure S11. PL spectra of ICzDO and ICzDM measured at room temperature (RT) and low temperature (LT).

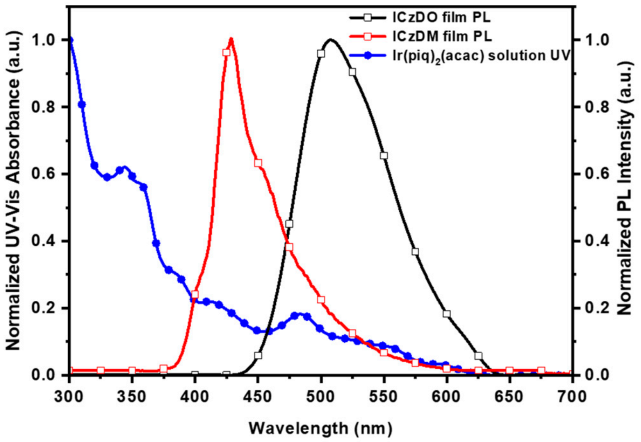

Figure S12. Overlap of the emission spectrum of Ir(piq)$_2$(acac) with the absorption spectra of the newly synthesized materials.
